# How reliably can northeast Atlantic sand lances of the genera *Ammodytes* and *Hyperoplus* be distinguished? A comparative application of morphological and molecular methods

**DOI:** 10.3897/zookeys.617.8866

**Published:** 2016-09-15

**Authors:** Ralf Thiel, Thomas Knebelsberger

**Affiliations:** 1 University of Hamburg, Center of Natural History, Zoological Museum, 20146 Hamburg, Germany; 2 Senckenberg am Meer, German Centre for Marine Biodiversity Research (DZMB), 26382 Wilhelmshaven, Germany

**Keywords:** Ammodytes, *COI*, DNA barcoding, Hyperoplus, meristic characters, mitochondrial DNA, morphology, morphometrics, northeast Atlantic, nuclear gene, Rhodopsin, Sand lances, species identification

## Abstract

Accurate stock assessments for each of the dominant species of sand lances in the northeast Atlantic Ocean and adjacent areas are not available due to the lack of a reliable identification procedure; therefore, appropriate measures of fisheries management or conservation of sand lances cannot be implemented. In this study, detailed morphological and molecular features are assessed to discriminate between four species of sand lances belonging to the genera *Ammodytes* and *Hyperoplus*.

Morphological characters described by earlier authors as useful for identification of the genera are confirmed, and two additional distinguishing characters are added. A combination of the following morphological characters is recommended to distinguish between the genera *Hyperoplus* and *Ammodytes*: the protrusibility of the premaxillae, the presence of hooked ends of the prevomer, the number of dermal plicae, and the pectoral-fin length as a percentage of the standard length. The discriminant function analysis revealed that morphometric data are not very useful to distinguish the species of each of the two genera. The following meristic characters improve the separation of *Hyperoplus lanceolatus* from *Hyperoplus immaculatus*: the number of lower arch gill rakers, total number of gill rakers, numbers of caudal vertebrae and total vertebrae, and numbers of dorsal-fin and anal-fin rays. It is confirmed that *Ammodytes tobianus* differs from *Ammodytes marinus* by its belly scales that are organised in tight chevrons, scales which are present over the musculature at the base of the caudal fin, as well as by the lower numbers of dermal plicae, dorsal-fin rays, and total vertebrae.

In contrast to the morphological data, mitochondrial *COI* sequences (DNA barcodes) failed to separate unambiguously the four investigated species. *Ammodytes tobianus* and *Hyperoplus lanceolatus* showed an overlap between intraspecific and interspecific K2P genetic distances and cannot be reliably distinguished using the common DNA barcoding approach. *Ammodytes marinus* and *Hyperoplus immaculatus* exhibited gaps between intraspecific and interspecific K2P distances of 2.73 and 3.34% respectively, indicating that their DNA barcodes can be used for species identification. As an alternative, short nuclear Rhodopsin sequences were analysed and one diagnostic character was found for each of the species *Ammodytes marinus*, *Hyperoplus lanceolatus*, and *Hyperoplus immaculatus*. *Ammodytes tobianus* can be characterised by the lack of species-specific mutations when compared to the other three species. In contrast to *COI*, the short nuclear sequences represent a useful alternative for rapid species identification whenever an examination of morphological characters is not available.

## Introduction

Sand lances of the family Ammodytidae are small fishes that live primarily in marine and adjacent brackish waters with sandy substrates of the northern hemisphere, where they are able to quickly dive into the substrate to escape predation (Randall and Ida 2014, Orr et al. 2015). These fishes are characterised by elongated and subcylindrical bodies and possess relatively low elongated dorsal and anal fins without spines, which are separated from the forked caudal fin (e.g. Reay 1986). The number of principal caudal rays is reduced and there is no pelvic fin in most species (e.g. Ida et al. 1994). Sand lances have an increased number of vertebrae in which the number of pre-caudal vertebrae is higher than the number of caudal vertebrae. The lower jaws project beyond the upper jaws. Small and unobtrusive scales are present (e.g. Reay 1986) and the body is often covered in oblique skinfolds (so-called plicae).

The family Ammodytidae comprises 31 species in seven genera (e.g. Randall and Ida 2014, Orr et al. 2015) of which the two genera *Ammodytes* and *Hyperoplus* are distributed circumboreally (Ida et al. 1994). Five species of sand lances belonging to three genera occur in northeast Atlantic waters (Sparholt 2015). This includes the Common sand eel *Ammodytes tobianus* Linnaeus, 1758 and the Lesser sand eel *Ammodytes marinus* Raitt, 1934, currently recognised together with four further species in the genus *Ammodytes* (Orr et al. 2015). Additionally, both species of the genus *Hyperoplus*, Corbin´s sand eel *Hyperoplus immaculatus* (Corbin, 1950) and the Greater sand eel *Hyperoplus lanceolatus* (Le Sauvage, 1824), can be found in the eastern north Atlantic area (Reay 1986), as well as *Gymnammodytes semisquamatus* (Jourdain, 1879). The latter can morphologically be distinguished from the species mentioned above by having a branched lateral line, a body not covered in oblique plicae (Cameron 1959), and scales that are loosely scattered and restricted to the posterior third of the body (Reay 1986), whereas the genera *Hyperoplus* and *Ammodytes* exhibit plicae along the body and an unbranched lateral line.

In identification keys these two genera are often distinguished by showing clear protrusible premaxillae and no vomerine teeth (*Ammodytes*) or no clear protrusible premaxillae and a pair of vomerine teeth (*Hyperoplus*, e.g. Reay 1986). *Hyperoplus lanceolatus* can be separated from *Hyperoplus immaculatus* by the occurrence of a conspicuous dark spot on either side of the snout below the anterior nostril. This spot is lacking in *Hyperoplus immaculatus*. *Ammodytes tobianus* is generally distinguished from *Ammodytes marinus* by its characteristic belly scales that are organised in tight chevrons and having scales present over the musculature at the base of the caudal fin, whereas these features are not present in *Ammodytes marinus* (Reay 1986).

However, the distinguishing features mentioned above are not easy to observe for the untrained eye when comparative material of different species is not available. Furthermore, an accurate species identification, especially of juvenile individuals, is difficult and even sub-adult and adult sand lances are difficult to identify (Sparholt 2015), if identification procedure is restricted to the few morphological characters mentioned above. In this context, Naevdal and Thorkildsen (2002) mentioned the difficulties regarding morphological separation of some of the five species of sand lances found in the northeast Atlantic and suggested a method for successful species identification on the basis of allozyme variation. DNA restriction fragment patterns have also been proposed to distinguish between some of the Atlantic sand eel species (Mitchell et al. 1998) as an alternative to morphological characters.

The difficult identification of sand lance species contributed to the current situation that accurate stock assessments are not available separately for each of the species in the North Sea and adjacent areas (see Sparholt 2015). However, sand lances here are subject to large-scale, industrial exploitation for fish meal and oil production and are also a major prey for many predators such as piscivorous fish, birds, and mammals (e.g. Reay 1986). It is known that exploitation of sand lances affects the food availability for these predators and that the abundance of sand lances is sensitive to recruitment variation (Sparholt 2015). Sand lances are divided into seven stock components for stock assessments in the North Sea based on the most abundant species *Ammodytes marinus*. With this approach, the stock situation of the single species cannot be evaluated, as it does not consider that sand lances represent a mix of different species. Clearly, another drawback is that an evaluation of the conservation status of the single species of sand lances is not possible (Thiel et al. 2013).

Molecular-based identification methods of fish species have been developed over the last decades (for an overview see Teletchea 2009). In this context, DNA barcoding constitutes the most popular and effective technique by using partial cytochrome *c* oxidase subunit I (COI) sequences for a standardised and routine identification of specimens to species level (Hebert et al. 2003). For a successful application of DNA barcoding as a tool for specimen identification, reliable sequence reference libraries such as the Barcode of Life Database (BOLD, Ratnasingham and Hebert 2007) were developed. Newly generated DNA barcodes can be uploaded and analysed together with data already available on BOLD in order to provide taxonomic identification. Additionally, barcode sequences were automatically analysed on BOLD and a Barcode Index Number (BIN) is assigned according to the calculated sequence clusters (Ratnasingham and Hebert 2013). Taxonomic conflicts apparently occur if sequences assigned to the same species name can be found within different BIN clusters.

For fish, the species discrimination success of DNA barcoding was demonstrated in many studies including freshwater as well as marine faunas from many regions all over the world (e.g. Ward et al. 2005, Hubert et al. 2008, Ward et al. 2008a, Steinke et al. 2009, April et al. 2011, Mabragaña et al. 2011, Costa et al. 2012, Zhang and Hanner 2012, Keskin and Atar 2013, McCusker et al. 2013, Geiger et al. 2014, Knebelsberger et al. 2014, Knebelsberger and Thiel 2014, Knebelsberger et al. 2015). DNA barcodes have also been successfully used to identify fish larvae (Pegg et al. 2006, Victor et al. 2009, Hubert et al. 2010, Kim et al. 2010), and fins (Holmes et al. 2009), and can provide evidence for cryptic diversity (Hubert et al. 2010, Ward et al. 2008b, Zemlak et al. 2009, Puckridge et al. 2013, Geiger et al. 2014, Knebelsberger et al. 2015).

For the North Sea and adjacent areas, two DNA barcoding studies revealed successful differentiation of all investigated species (Knebelsberger et al. 2014, Knebelsberger and Thiel 2014). Altogether, 105 species belonging to 88 genera were analysed. Most of the genera were represented by only one species. As an exception, the genus *Pomatoschistus* was represented by five closely related species.

One of these studies already provided DNA barcodes for the two sand lance species *Ammodytes marinus* and *Hyperoplus immaculatus* and demonstrated a clear separation of these two species (Knebelsberger et al. 2014). A former study from continental Portugal Atlantic waters included DNA barcodes for *Hyperoplus lanceolatus* but other species of sand lances were missing (Costa et al. 2012). Studies including congeneric species of the genus *Ammodytes* revealed inconsistencies between morphological and DNA barcode-based identification: for two species from the northwest Atlantic Ocean, namely *Ammodytes americanus* and *Ammodytes dubius*, barcoding fails to separate these species, which may be caused by inadequate taxonomy (McCusker et al. 2013). Inadequate taxonomy may also concern the two species *Ammodytes personatus* and *Ammodytes hexapterus* from the north Pacific (Turanov and Kartavtsev 2014). In both cases the taxonomic status of the species is questionable and may require comprehensive taxonomic revision. In order to examine the application of DNA barcoding for the identification of sand lances from the North Sea area, all closely related species from this region must be included. This concerns *Ammodytes marinus* and *Hyperoplus immaculatus* as well as *Ammodytes tobianus* and *Hyperoplus lanceolatus*. For the latter two species reliable *COI* data from the North Sea are still missing.

This paper presents the first comprehensive study combining morphological and molecular methods for the discrimination of four species of sand lances belonging to the genera *Ammodytes* and *Hyperoplus* occurring in the northeast Atlantic Ocean and adjacent waters. The suitability of two morphological types of parameters (meristic characters and morphometric measurements) and two genetic approaches (mitochondrial *COI* (DNA barcoding region) and partial nuclear Rhodopsin DNA sequences) for accurate species identification is examined. A detailed and accurate species identification matrix is presented, based on the integration of morphological and molecular traits.

## Materials and methods

### Material

**Figure 1.**
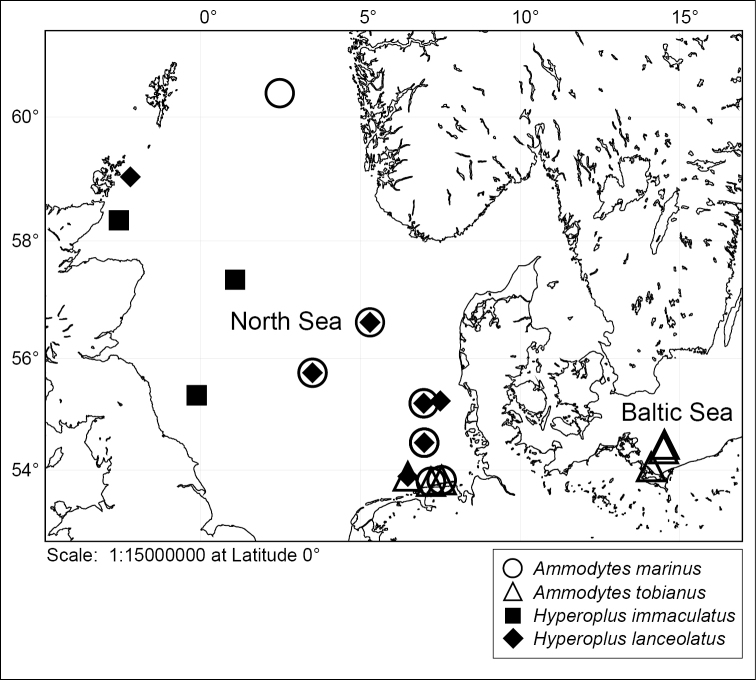
Sampling sites of sand lance species of the genera *Ammodytes* and *Hyperoplus*.

In this study 85 specimens representing two species of genus *Ammodytes* and two species of genus *Hyperoplus* were sampled from the North and the Baltic Seas (Suppl. material 1 and 2, Figure 1). For the molecular analysis 70 samples were collected from the North Sea during several cruises conducted by the Thünen Institute of Sea Fisheries (Hamburg, Germany) and the research vessel of the Senckenberg Institute (Wilhelmshaven, Germany). Tissue samples were taken from each of the 70 specimens and preserved in 96% ethanol for molecular analysis at Senckenberg’s German Center for Marine Biodiversity Research (DZMB, Wilhelmshaven, Germany). Specimens were preserved in 70% ethanol. The remaining 15 individuals belonging to the species *Ammodytes tobianus* were used for morphological analyses only and collected from the Baltic Sea during three different cruises conducted by the German Oceangraphic Museum (Stralsund, Germany). Immediately after catch, specimens were preserved in 4% formaldehyde solution. All 85 voucher specimens were databased and morphologically investigated at the Zoological Museum of the Center of Natural History of the University of Hamburg (ZMH, Hamburg, Germany). Finally, the material was stored for future reference in the ZMH fish collection. All *COI* sequences and related metadata belonging to the 70 voucher specimens from the North Sea are available on the Barcode of Life Data System (www.barcodinglife.org; Ratnasingham and Hebert 2007). DNA barcodes of eight specimens of *Hyperoplus immaculatus* and 22 specimens of *Ammodytes marinus* were obtained from the BOLD project “Barcoding North Sea Fish I” (BNSFI) (Knebelsberger et al. 2014). Newly generated barcodes belonging to five specimens of *Ammodytes marinus*, six of *Ammodytes tobianus*, and 29 of *Hyperoplus lanceolatus* were uploaded to the BOLD project “Barcoding North Sea Sand eels” (BNSSE). In addition to *COI*, nuclear Rhodopsin DNA sequences were generated from all 70 North Sea specimens (Suppl. material 1). For comparison, published Rhodopsin data was downloaded from GenBank (*Ammodytes tobianus*: AY141306; *Hyperoplus lanceolatus*: EU492010 and EU492011).

### Morphological analyses

**Figure 2.**
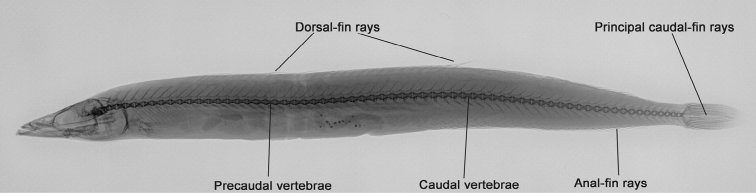
Radiograph of Common sand eel *Ammodytes tobianus* (Linnaeus, 1758) indicating the meristic characters evaluated from X-ray pictures. Depicted specimen: ZMH 26098-3, standard length 128.1 mm.

Meristic parameters (Table 1) were analysed at the left-hand side of the specimens and supplemented with right-side counts when the left side was damaged. Counts of dorsal, ventral and principal caudal-fin rays as well as of vertebrae were taken from radiographs (Figure 2) made by an X-ray imaging system (Faxitron LX-60). The first caudal vertebra was defined as the first centrum with a long haemal spine, and the centrum fused to the hypural plate was counted as the last vertebra. Counts of dorsal-fin rays were made using the method of Nizinski et al. (1990). Counting dorsal-fin rays began with the first visible ray and excluded the one or two anterior rayless pterygiophores. However, these counts included the last two rays that were each supported by a pterygiophore. Counts of anal-fin rays included all rays visible from the outside. Gill rakers were counted on the lower and upper arch separately. Gill rakers of the lower arch included the raker at the junction between upper and lower parts of the arch. Dermal plicae included those anterior and posterior to the lateral-line pores.

**Table 1.** Data of estimated morphological characters of four species of sand lances of the genera *Ammodytes* and *Hyperoplus*. If possible, each meristic character and morphometric measurement is presented with its range (before the semicolon), mean value with standard deviation and the number of specimens analysed (in brackets). Morphometric measurements are given as proportion of SL.

Species	*Ammodytes marinus*	*Ammodytes tobianus*	*Hyperoplus immaculatus*	*Hyperoplus lanceolatus*
Meristic characters				
Dermal plicae (DP)	140–150; 143.1 ± 2.6 (24)	123–135; 128.5 ± 3.0 (21)	179–196; 187.8 ± 5.4 (8)	169–194; 182.6 ± 6.4 (29)
Dorsal-fin rays (DR)	56–62; 59.1 ± 1.4 (27)	51–55; 52.8 ± 1.1 (21)	59–61; 60.1 ± 0.6 (8)	54–57; 55.9 ± 0.8 (29)
Anal-fin rays (AR)	28–31; 29.5 ± 0.8 (27)	25–30; 27.8 ± 1.3 (21)	31–32; 31.8 ± 0.5 (8)	28–30; 29.1 ± 0.8 (29)
Pectoral-fin rays (PR)	12–15; 13.6 ± 0.6 (26)	12–13; 13.0 ± 0.2 (21)	13–15; 14.1 ± 0.6 (8)	13–14; 13.5 ± 0.5 (29)
Principal caudal-fin rays (CR)	15 ± 0 (27)	15 ± 0 (21)	15 ± 0 (8)	15 ± 0 (29)
Upper arch gill rakers (UR)	5–6; 5.0 ± 0.2 (24)	5 ± 0 (21)	5 ± 0 (8)	5 ± 0 (29)
Lower arch gill rakers (LR)	18–20; 18.6 ± 0.8 (24)	20 ± 0 (21)	23–25; 24.3 ± 0.7 (8)	20–22; 20.7 ± 0.8 (29)
Total gill rakers (GR)	23–26; 23.6 ± 0.9 (24)	25 ± 0 (21)	28–30; 29.1 ± 0.6 (8)	25–27; 25.7 ± 0.8 (29)
Precaudal vertebrae (PV)	42–44; 42.9 ± 0.7 (27)	36–41; 38.1 ± 1.4 (21)	42–43; 42.9 ± 0.4 (8)	38–42; 40.3 ± 0.8 (29)
Caudal vertebrae (CV)	26–28; 26.7 ± 0.5 (27)	24–26; 25.1 ± 0.6 (21)	29–30; 29.5 ± 0.5 (8)	25–28; 26.5 ± 0.7 (29)
Total vertebrae (TV)	68–71; 69.6 ± 0.9 (27)	60–66; 63.2 ± 1.4 (21)	72–73; 72.4 ± 0.5 (8)	65–68; 66.8 ± 0.8 (29)
Margins of dorsal and anal fins straight with rays of equal length (MDAS)	yes (27)	yes (21)	yes (8)	yes (29)
Body covered in oblique plicae bearing the scales (BCOP)	yes (27)	yes (21)	yes (8)	yes (29)
Premaxillae clearly protrusible (PCP)	yes (27)	yes (21)	no (8)	no (29)
Two vomerine teeth present (VTP)	no (27)	no (21)	yes (8)	yes (29)
Conspicuous dark spot on either side of snout (DSSS)	no (27)	no (21)	no (8)	yes (29)
Belly scales in tight chevrons (BSTC)	no (27)	yes (21)	yes (8)	not clearly detectable (29)
Scales over musculature at base of caudal fin (SBCF)	no (27)	yes (21)	yes (8)	yes (29)
Scales present in the midline anterior to dorsal fin (SADF)	no (27)	yes (21)	yes (8)	yes (29)
Morphometric measurements				
Standard length (SL, mm)	61.2–193; 136.7 ± 29.7 (27)	121.5–146.4; 134.1 ± 7.7 (21)	220.4–270.1; 251.4 ± 15.0 (8)	165.0–291.0; 219.3 ± 32.7 (29)
Percentage standard length				
Body depth at dorsal-fin origin (BDD)	5.9–11.1; 9.1 ± 1.3 (26)	8.3–11.9; 9.5 ± 0.8 (21)	5.8–7.9; 6.8 ± 0.9 (8)	5.6–8.8; 7.0 ± 0.9 (29)
Body depth at anal-fin origin (BDA)	4.9–10.1; 8.1 ± 1.2 (27)	7.3–10.4; 9.4 ± 0.8 (20)	6.6–8.2; 7.5 ± 0.5 (8)	5.2–8.0; 6.7 ± 0.6 (28)
Body with at dorsal-fin origin (BWD)	4.1–6.4; 5.3 ± 0.7 (26)	4.0–7.6; 6.0 ± 0.9 (21)	5.6–8.2; 6.5 ± 0.8 (8)	4.3–7.2; 5.4 ± 0.8 (29)
Head length (HL)	17.6–22.6; 20.0 ± 1.1 (26)	18.4–21.4; 19.6 ± 0.8 (21)	18.3–20.2; 19.4 ± 0.7 (8)	20.2–23.0; 21.7 ± 0.6 (29)
Snout length (SNL)	5.1–6.1; 5.7 ± 0.3 (26)	5.1–6.0; 5.4 ± 0.3 (21)	6.0–6.3; 6.1 ± 0.1 (8)	5.0–8.1; 7.2 ± 0.5 (29)
Orbit diameter (OD)	2.7–4.4; 3.2 ± 0.5 (26)	2.6–3.5; 3.0 ± 0.3 (21)	1.9–2.5; 2.3 ± 0.2 (8)	2.0–4.5; 2.5 ± 0.5 (29)
Interorbital width (IW)	2.0–3.5; 2.4 ± 0.3 (27)	2.1–2.9; 2.4 ± 0.2 (21)	2.8–3.6; 3.2 ± 0.3 (8)	2.7–3.7; 3.3 ± 0.3 (29)
Upper jaw length (UJL)	4.6–10.3; 6.7 ± 1.0 (26)	5.4–7.0; 6.4 ± 0.4 (21)	4.7–6.0; 5.3 ± 0.4 (8)	6.5–8.1; 7.1 ± 0.3 (29)
Caudal peduncle depth (CPD)	2.2 -2.9; 2.6 ± 0.2 (26)	2.8–3.2; 3.0 ± 0.1 (21)	2.5–2.9; 2.7 ± 0.1 (8)	2.3–2.8; 2.5 ± 0.1 (29)
Caudal peduncle length (CPL)	3.2–5.6; 4.1 ± 0.5 (27)	3.5–5.4; 4.3 ± 0.5 (21)	4.4–6.4; 4.8 ± 0.7 (8)	3.9–5.7; 4.9 ± 0.4 (29)
Prepectoral length (PPL)	16.6–21.7; 18.6 ± 1.0 (25)	14.8–20.4; 18.6 ± 1.1 (21)	17.9–18.7; 18.4 ± 0.3 (8)	19.5–22.1; 20.8 ± 0.7 (29)
Predorsal length (PDL)	23.5–28.1; 25.3 ± 1.0 (26)	24.0–27.3; 25.4 ± 1.0 (21)	26.1–27.6; 26.6 ± 0.5 (8)	25.7–30.4; 27.9 ± 1.0 (29)
Preanal length (PAL)	63.4–66.8; 64.9 ± 1.1 (27)	60.3–67.0; 63.7 ± 1.9 (21)	60.2–64.1; 62.8 ± 1.4 (8)	45.4–70.6; 64.9 ± 4.1 (29)
Pectoral-fin length (PFL)	8.6–11.9; 9.9 ± 0.7 (25)	9.4–11.4; 10.4 ± 0.5 (21)	7.2–8.2; 7.7 ± 0.4 (8)	6.7–8.7; 7.7 ± 0.6 (28)
Dorsal-fin base length (DFBL)	66.6–72.0; 69.8 ± 1.4 (27)	66.9–70.8; 69.2 ± 1.1 (21)	68.1–69.8; 69.1 ± 0.7 (8)	65.5–69.2; 67.4 ± 1.1 (29)
Anal-fin base length (AFBL)	28.9–32.4; 30.5 ± 1.0 (27)	26.7–33.4; 31.6 ± 1.9 (21)	30.5–33.8; 32.4 ± 1.2 (8)	28.4–32.1; 29.8 ± 1.0 (29)
Caudal-fin length (CFL)	8.3–11.5; 10.1 ± 0.7 (26)	9.0–12.0; 10.3 ± 0.7 (21)	8.5–9.7; 8.9 ± 0.4 (8)	7.6–10.3; 8.9 ± 0.7 (28)
Dorsal-fin height (DFH)	3.6–6.0; 4.7 ± 0.7 (27)	3.9–6.1; 4.8 ± 0.6 (21)	3.4–4.6; 4.1 ± 0.4 (8)	2.7–5.7; 3.9 ± 0.6 (29)
Anal-fin height (AFH)	2.6–6.2; 4.7 ± 0.8 (27)	3.4–5.8; 4.8 ± 0.5 (21)	2.5–4.6; 3.9 ± 0.7 (8)	2.7–5.6; 4.1 ± 0.6 (29)

Morphometric measurements (Table 2) were taken by vernier calipers to one tenth of a millimetre. Measurements were done following Hubbs and Lagler´s (1958) method, with the following changes: standard length (SL) was measured from the front of the upper lip in the median plane to the midbase of the caudal fin (end of hypural plate). The front of the upper lip was used as the anterior point of all other horizontal measurements. Head length (HL) was measured from the front of the upper lip to the posterior end of the opercular membrane. Body depth was measured twice, as the depth at the beginning of the base of the dorsal fin (BDD) and as the depth at the beginning of the base of the anal fin (BDA). Body width was measured as the maximum width at the beginning of the base of the dorsal fin (BWD). Orbit diameter (OD) is the maximum fleshy diameter. Interorbital width (IW) is the least fleshy width. Caudal-peduncle depth (CPD) is the smallest depth, and caudal peduncle length (CPL) the horizontal distance between verticals at the rear base of the anal fin and the caudal-fin base. Height of dorsal-fin rays (DFH) and anal-fin rays (AFH) were measured from their tips to the body contour. Caudal-fin length (CFL) was taken horizontally from the caudal-fin base to a vertical point at the tip of the longest ray. The values obtained were standardised by multiplying them by 100 and dividing them by the standard length.

**Table 2.** Standardised coefficients of the first three discriminant functions (DF1, DF2, DF3) separating the four species of *Ammodytes* and *Hyperoplus* based on meristic characters. In bold, characters with the greatest weight in DF1 and DF2.

Meristic characters	DF1	DF2	DF3
DP	**0.884**	-0.320	-0.444
DR	0.056	**0.664**	-0.096
AR	0.112	-0.155	0.159
PR	0.015	0.079	-0.172
UR	-0.428	-0.135	-0.289
LR	**0.432**	-0.140	0.891
PV	-0.017	**0.523**	-0.061
CV	0.426	0.516	0.471
Percentage of explained variance	71.438	20.317	8.245
Eigenvalue	40.392	11.488	4.662
Cumulative variance in %	71.438	91.755	100.00

### Statistical treatment of morphological data

All morphological data were statistically processed, involving ranges, means, and standard deviations. Morphological data of all specimens that had a complete suite of meristic and morphometric character data were used to conduct two multiple discriminant function analyses (DFA) to determine if the four species of sand lances could be differentiated based on meristic and/or morphometric parameters using XLSTAT (version 2013.0.04, Addinsoft), a statistical analysis add-in for Microsoft Excel®. DFA was used to demonstrate the degree of separation in multivariate space defined by the main patterns of morphological variation among species which is described via the discriminant functions. It also shows which character contributes more to the differentiation. The standardised discriminant function coefficients represent the contributions of every variable to the discriminatory power of the function. Hence, the larger the standardised coefficient, the larger the weight of the variable in the function. Both discriminant analyses were conducted for 76 individuals (22 *Ammodytes marinus*, 20 *Ammodytes tobianus*, 8 *Hyperoplus immaculatus*, 26 *Hyperoplus lanceolatus*). Morphological variables without any variation (e.g. principal caudal-fin rays (CR)), variables, where other variables are included (e.g. total vertebrae (TV)) and qualitative variables (e.g. premaxillae clearly protrusible (PCP)) were excluded from the DFA procedures. The first DFA was performed for the following eight quantitative meristic characters: dermal plicae, dorsal-fin rays, anal-fin rays, pectoral-fin rays, upper arch gill rakers, lower arch gill rakers, precaudal vertebrae, and caudal vertebrae. The second DFA was conducted for the following 19 morphometric parameters: body depth at dorsal-fin origin, body depth at anal-fin origin, body width at dorsal-fin origin, head length, snout length, orbit diameter, interorbital width, upper jaw length, caudal peduncle depth, caudal peduncle length, prepectoral length, predorsal length, preanal length, pectoral-fin length, dorsal-fin base length, anal-fin base length, caudal-fin length, dorsal-fin height, and anal-fin height.

### DNA isolation, PCR amplification, and sequencing

Genomic DNA was extracted at the DZMB using the Qiagen DNeasy Blood and Tissue Kit for single columns as described by Knebelsberger and Stöger (2012). A 652-bp fragment of the mitochondrial (mt) *COI* gene was amplified for 38 samples using a M13 tailed primer cocktail (C_FishF1t1-C_FishR1t1) including FishF2_t1 (5’-TGTAAAACGACGGCCAGTCGACTAATCATAAAGATATCGGCAC), FishR2_t1 (5’-CAGGAAACAGCTATGACACTTCAGGGTGACCGAAGAATCAGAA), VF2_t1 (5’-TGTAAAACGACGGCCAGTCAACCAACCACAAAGACATTGGCAC) and FR1d_t1 (5’-CAGGAAACAGCTATGACACCTCAGGGTGTCCGAARAAYCARAA) (Ivanova et al. 2007). As a second marker, a 464 bp fragment of the nuclear (nc) Rhodpsin gene was amplified for all 70 samples using the forward primer Rh545 (5’-GCAAGCCCATCAGCAACTTCCG) and the reverse primer Rh1039r (5’-TGCTTGTTCATGCAGATGTAGA) developed by Chen et al. (2003). Each PCR reaction mixture contained 1 µl DNA template, 2.25 µl of 10X reaction buffer (including MgCl_2_), 0.5 µl dNTPs (2mM each), 0.5 µl of each primer (10 pmol/µl), 0.5 µl Taq polymerase (5 U/µl; Qiagen) and molecular grade water for a total volume of 25µl. Thermal cycling was performed with an initial denaturation for 2 min at 94°C, followed by 35 cycles of 30 s at 94°C, 30 s at the annealing temperature of 50°C, 60 s at 72°C with a final extension of 10 min at 72°C. All PCR products were checked by a 1% agarose gel. Amplicons were purified by incubating 10 µl of PCR products with 0.5 µl of Exonuclease I (20 U/µl) and 2 µl of Alkaline Phosphatase (1 U/µl) for 15 min at 37°C followed by 20 min at 75°C. Purified amplicons were sequenced by Macrogen Europe (Amsterdam, Netherlands).

### Sequence alignment and data analyses

Forward and reverse sequences of *COI* and Rhodpsin were assembled and edited using Geneious (version 7.1.9. http://www.geneious.com). Consensus sequences were submitted to GenBank (for accession numbers see Suppl. material 1). Variance in sequence length, base composition, number of invariable sites and the presence of stop codons were analysed using Geneious. The nc and mt sequences were aligned independently using MUSCLE (Edgar 2004) with default settings as implemented in MEGA version 6.06 (Tamura et al. 2013). Primer sequences were cut from the alignment. Rhodopsin gene sequence alignment was checked by eye for species specific diagnostic characters. For *COI*, Kimura-2-parameter (K2P) distances were calculated in MEGA, as K2P is used as standard model for barcoding analyses and enables direct comparison with other studies. Neighbour-Joining (NJ) topology (Saitou and Nei 1987) was built in MEGA using the “pairwise deletion” option for the treatment of gaps and missing data, in order to retain all sites initially, excluding them as necessary. Node support for the NJ topology was evaluated by a non-parametric bootstrap analysis (Felsenstein 1985) with 10,000 replicates. In order to quantify the distinctness between species at the barcode locus, genetic distances were used to calculate the difference between the maximum intraspecific genetic distance and the minimum distance to the nearest neighbor (barcode gap). For the calculation of genetic distances at genus and family level, we used BOLDs “Distance Summary” tool by choosing K2P distance model and MUSCLE (Edgar 2004) alignment algorithm.

On BOLD, DNA barcodes were automatically assigned to operational taxonomic units (OTUs), generated through Refined Single Linkage (RESL) analyses (Ratnasingham and Hebert 2013). Finally, a unique alphanumeric code is assigned to each of the OTUs, constituting the so called barcode index number (BIN). It has been shown that BINs are highly congruent with existing species assignments (Ratnasingham and Hebert 2013). Here, the ‘BIN Discordance Report’ analysis tool was applied to analyse our dataset together with public sequences on BOLD, and to get hints on cryptic diversity (species) or to identify cases of haplotype sharing between species.

Furthermore, BOLD’s “Diagnostic Characters” sequence analysis tool was applied to the COI dataset choosing MUSCLE (Edgar 2004) alignment algorithm. Sequences were grouped by species names in order to categorise consensus bases by their diagnostic potential.

## Results

### General results of morphological analysis

Meristic characters and morphometric measurements of the four examined species of sand lances are given in Table 1. The number of individuals per analysed character ranged from 24 to 27 for *Ammodytes marinus*, from 20 to 21 for *Ammodytes tobianus*, from 28 to 29 for *Hyperoplus lanceolatus*, and was eight individuals for *Hyperoplus immaculatus* (Table 1).

The data of the present study confirmed that the two genera of *Ammodytes* and *Hyperoplus* can be distinguished by qualitative meristic characters, i.e. by having a clear protrusible premaxillae (PCP) and no vomerine teeth (VTP) (*Ammodytes*) or no clear protrusible premaxillae and two vomerine teeth (*Hyperoplus*) (Table 1). *Hyperoplus* can also be separated from *Ammodytes* by its significantly higher number of dermal plicae (DP). It is also possible to distinguish *Hyperoplus* from *Ammodytes* by its obviously lower pectoral-fin length (PFL), and to a somewhat lesser significance, also by its greater mean snout length (SNL), since no sexual dimorphism has been reported for the last two characters in both genera.

*Hyperoplus lanceolatus* can be separated from *Hyperoplus immaculatus* by the presence of a conspicuous dark spot on either side of snout (DSSS) which is lacking in *Hyperoplus immaculatus* (Table 1). Furthermore, *Hyperoplus lanceolatus* differs from *Hyperoplus immaculatus* by its lower numbers of total and lower arch gill rakers (GR, LR), total and caudal vertebrae (TV, CV), as well as dorsal and anal-fin rays (DR, AR).

*Ammodytes tobianus* can be distinguished from *Ammodytes marinus* by having belly scales that are organised in tight chevrons (BSTC) and having scales present over the musculature at the base of the caudal fin (SBCF) and in the midline anterior to dorsal fin (SADF), whereas these characters are not present in *Ammodytes marinus* (Table 1). *Ammodytes tobianus* differs from *Ammodytes marinus* also by its lower numbers of dermal plicae (DP), dorsal-fin rays (DR), and precaudal and total vertebrae numbers (PV, TV).

### Discriminant Function Analysis with meristic characters

DFA based on meristic characters provided three significant functions (Box-Test with χ^2^ = 790.916 and p<0.0001; Wilks´ lambda = 0.0003 and p<0.0001). These three functions explain 100% of the total variation in the data. The first two functions explain 91,755% of the total variation in the data (Table 2), which is sufficient for the further detailed analysis. The third discriminant function explains 8.245% of total variation.

**Figure 3.**
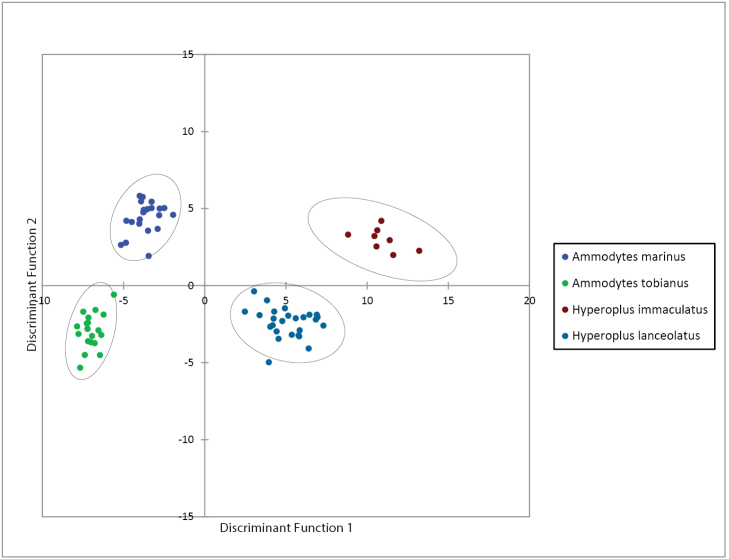
Plot of all analysed *Ammodytes* and *Hyperoplus* specimens onto the first and second discriminant functions based on a set of eight meristic characters. Circles include 95% of specimens in each species.

Individual specimens are projected onto the first two discriminant functions in Figure 3. Because all four species were clearly separated in the discriminant space defined by the first two functions, the third function was not used. The first discriminant function explains 71.438% of total variation (Table 2). It mainly separates *Ammodytes tobianus* and *Hyperoplus immaculatus* and to a lesser extent the species pairs of *Ammodytes tobianus* and *Hyperoplus lanceolatus*, *Ammodytes marinus* and *Hyperoplus immaculatus*, *Ammodytes marinus* and *Hyperoplus lanceolatus* as well as *Hyperoplus lanceolatus* and *Hyperoplus immaculatus* (Figure 3). *Ammodytes tobianus* and *Ammodytes marinus* cannot be so clearly separated by the first discriminant function.

From the standardised coefficients (Table 2), the two characters that have the greatest influence on the first discriminant function (characters most discriminatory) are the dermal plicae (DP) and lower arch gill rakers (LR) (Table 2). In general *Hyperoplus immaculatus* and *Hyperoplus lanceolatus* have a much higher number of dermal plicae than *Ammodytes tobianus* and *Ammodytes marinus* (Table 1). The numbers of DP of both species of *Hyperoplus* are overlapping, whereas the *Ammodytes* species have different numbers of DP. The number of lower arch gill rakers (LR) is higher in *Hyperoplus immaculatus* in comparison with the other three species, which have overlapping numbers of LR.

The second discriminant function accounts for 20.317% of total variation. *Ammodytes tobianus* and *Ammodytes marinus* are clearly and the species pairs of *Ammodytes tobianus* and *Hyperoplus immaculatus*, *Ammodytes marinus* and *Hyperoplus lanceolatus* as well as *Hyperoplus immaculatus* and *Hyperoplus lanceolatus* are to a lesser extent discriminated by this function. *Ammodytes tobianus* and *Hyperoplus lanceolatus* and *Ammodytes marinus* and *Hyperoplus immaculatus* cannot be clearly separated by the second discriminant function. The contrasts between the numbers of dorsal-fin rays (DR) and the numbers of precaudal vertebrae (PV) of the species are mainly responsible for this discrimination. DR is lowest in *Ammodytes tobianus* and highest in *Hyperoplus immaculatus* (Table 1). PV is lowest in *Ammodytes tobianus* and highest in *Ammodytes marinus* and *Hyperoplus immaculatus*.

### Discriminant Function Analysis with morphometric measurements

Three significant DFA functions were estimated based on morphometric measurements (Box-Test with χ^2^ = 944.979 and p < 0.0001; Wilks´ lambda = 0.003 and p < 0.0001). Together these functions explain 100% of the total variation in the data. The first two functions explain 93.144% of the total variation in the data (Table 3), which is sufficient for the further detailed analysis. The third discriminant function explains 6.856% of total variation.

**Table 3.** Standardised coefficients of the first three discriminant functions (DF1, DF2, DF3) separating the four species of *Ammodytes* and *Hyperoplus* based on morphometric measurements. In bold, characters with the greatest weight in DF1 and DF2.

Morphometric measurements	DF1	DF2	DF3
BDD	-0.135	-0.184	0.085
BDA	-0.178	-0.038	-0.208
BWD	-0.054	0.317	0.222
HL	0.290	-0.084	-0.603
SNL	**0.380**	-0.040	-0.045
OD	-0.231	0.078	0.497
IW	0.198	0.164	0.237
UJL	-0.034	-**0.868**	-0.260
CPD	-0.228	**0.542**	-0.473
CPL	0.112	0.418	-0.147
PPL	0.204	0.101	-0.076
PDL	0.140	0.251	0.143
PAL	-0.020	0.008	0.103
PFL	-**0.612**	-0.280	-0.528
DFBL	-0.060	0.104	0.551
AFBL	0.098	0.429	-0.208
CFL	-0.148	0.520	0.390
DFH	-0.078	-0.125	0.223
AFH	0.136	-0.333	-0.259
Percentage of explained variance	78.576	14.568	6.856
Eigenvalue	20.555	3.811	1.794
Cumulative variance in %	78.576	93.144	100.00

**Figure 4.**
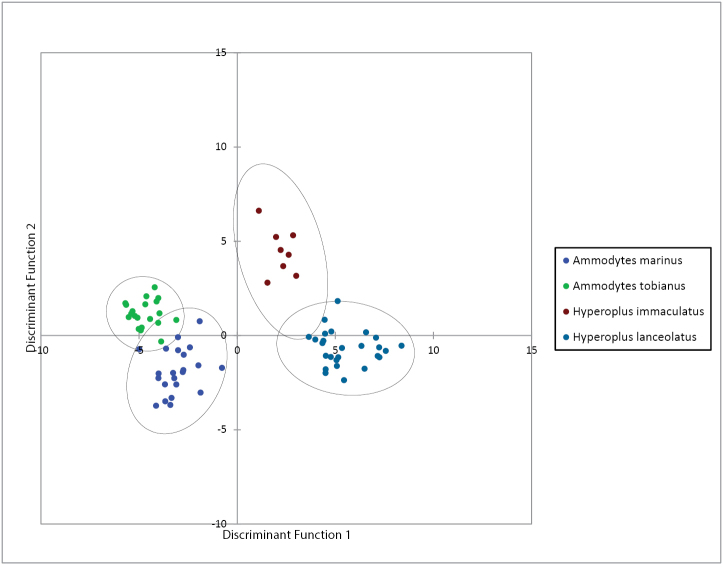
Plot of all analysed *Ammodytes* and *Hyperoplus* specimens onto the first and second discriminant functions based on a set of 19 morphometric characters. Circles include 95% of specimens in each species.

Figure 4 presents the individual specimens projected onto the first two discriminant functions. Because all four species were clearly separated in the discriminant space defined by the first two functions, the third function was not used. The first discriminant function explains 78.576% of total variation (Table 3). It mainly separates *Ammodytes tobianus* and *Hyperoplus lanceolatus* and to a lesser extent the species pairs of *Ammodytes tobianus* and *Hyperoplus immaculatus*, *Ammodytes marinus* and *Hyperoplus lanceolatus* as well as *Ammodytes marinus* and *Hyperoplus immaculatus* (Figure 4). The species pairs of *Ammodytes marinus* and *Ammodytes tobianus* as well as of *Hyperoplus immaculatus* and *Hyperoplus lanceolatus* cannot be separated by the first discriminant function.

The two measurement characters that have the greatest weight on the first discriminant function are pectoral-fin length (PFL) and the snout length (SNL) (Table 3). Both species of the genus *Ammodytes* have a greater PFL than both species of the genus *Hyperoplus* (Table 1). In contrast, both *Hyperoplus* species have a greater SNL than both *Ammodytes* species. PFL and SNL are relatively similar for the species of the same genera.

The second discriminant function accounts for 14.568% of total variation. Especially the species within the genera *Ammodytes* and *Hyperoplus*, namely *Ammodytes tobianus* and *Ammodytes marinus* as well as *Hyperoplus immaculatus and Hyperoplus lanceolatus* are separated by this function (Figure 4).


 Upper jaw length (UJL) and caudal peduncle depth (CPD) are the two measurements, for which no sexual dimorphism is known, and that have the greatest weight on the second discriminant function (Table 3).

### Mt DNA barcoding

Mitochondrial DNA barcodes were obtained for 70 specimens belonging to four species of the family Ammodytidae investigated in this study (Suppl. material 1). The DNA sequences did not show any ambiguous base calls (Ns) or stop codons, and no insertions or deletions were found within the sequence alignment. Sequence length ranged from 619 to 652 bp (mean and standard deviation: 650.5 ± 5.7 bp). The average base composition was 22.8% adenine (A), 29.7% cytosine (C), 18.3% guanine (G) and 29.3% thymine (T); GC content was 48%. The sequence alignment showed 588 identical sites.

**Figure 5.**
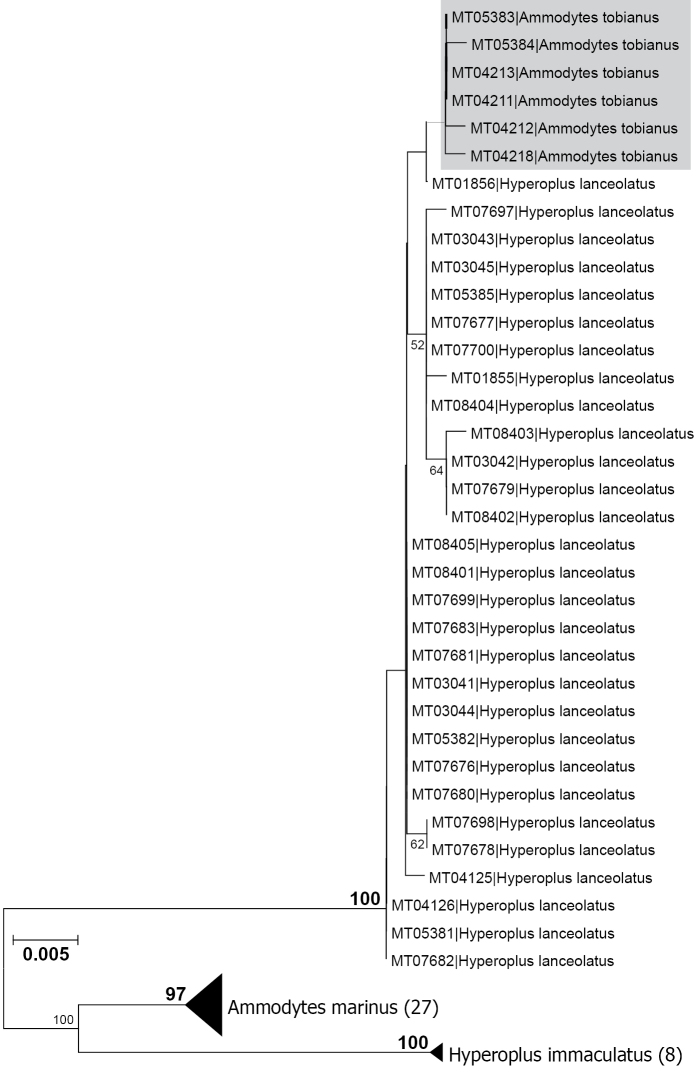
NJ dendrogram based on K2P pairwise genetic distances. Values at nodes indicate the result of the bootstrap test (10.000 pseudo replicates). Only values ≥ 50 are shown. For *Ammodytes tobianus* (grey box) and *Hyperoplus lanceolatus* all analysed individuals are shown. In case of *Ammodytes marinus* and *Hyperoplus immaculatus* the number of specimens is given in brackets.

The NJ analysis of the K2P distances revealed well supported monophyletic clusters for the species *Ammodytes marinus* and *Hyperoplus immaculatus* with bootstrap values of 97 and 100, respectively (Figure 5). In contrast, *Ammodytes tobianus* and *Hyperoplus lanceolatus* sequences were grouped together in one monophyletic cluster with a bootstrap support of 100. Within this cluster the sequences of *Ammodytes tobianus* were grouped together without bootstrap support indicating that there is no sharing of haplotypes between these two species. The analysis of the K2P genetic distances revealed an overlap between intraspecific (range: 0.0-0.77%; mean and standard deviation: 0.22 ± 0.17%) and interspecific distances (0.15-7.27%; 4.73 ± 1.7%). The overlap was caused by the two species *Ammodytes tobianus* and *Hyperoplus lanceolatus*: in *Ammodytes tobianus*, the minimum distance to the nearest neighbour species was even lower than the maximum intraspecific distance, whereas both values were equal in *Hyperoplus lanceolatus* (Table 4). In contrast, the species *Ammodytes marinus* and *Hyperoplus immaculatus* exhibited barcode gaps of 2.73% and 3.34% respectively, which indicates an undoubtedly separation from the other species. At genus and family level, the genetic distances between species of the same genus varied between 4.46-7.09% and the distances between species belonging to different ranged from 0.15-7.27%.

**Table 4.** Minimum and maximum intraspecific genetic K2P distances (%) for each species including mean and standard deviation. The barcoding gap indicates the difference between the maximum intraspecific and the minimum interspecific (nearest neighbour) genetic distance. Additionally K2P genetic distances are given in brackets, if they differ from p-distances.

Species	Specimens	Mean Distance	SD*	Minimum Distance	Maximum Distance	Nearest Neighbor	Distance to Nearest Neighbor	Barcoding Gap
*Ammodytes marinus*	27	0.24	0.19	0.00	0.77	*Hyperoplus immaculatus*	3.50	2.73
*Ammodytes tobianus*	6	0.15	0.01	0.00	0.15	*Hyperoplus lanceolatus*	0.15	no gap
*Hyperoplus lanceolatus*	29	0.22	0.15	0.00	0.62	*Ammodytes tobianus*	0.15	no gap
*Hyperoplus immaculatus*	8	0.07	0.08	0.00	0.16	*Ammodytes marinus*	3.50	3.34

### BIN report

The BIN discordance report tool on BOLD assigned three different BIN numbers to the 70 *COI* haplotypes. The BIN BOLD:ACF3320 was found to be “concordant” and exclusively comprised 32 specimens of the species *Ammodytes marinus*, of which five individuals were not provided by this study. The “discordant” BIN BOLD:AAC5676 comprised 57 specimens, 14 identified as *Ammodytes tobianus* and 43 as *Hyperoplus lanceolatus*. From the former species eight specimens and from the latter 14 specimens were not provided by our study but also support the findings of this study. The third BIN BOLD:AAJ2299 was also specified as discordant and comprised ten specimens, eight (in our study) identified as *Hyperoplus immaculatus* and two identified as *Ammodytes marinus*. The two *Ammodytes marinus* entries may represent cases of misidentification as 32 *Ammodytes marinus* individuals were grouped together in BIN BOLD:ACF3320.

### Diagnostic characters

The analysis revealed four diagnostic characters for the species *Ammodytes marinus* and 16 for *Hyperoplus immaculatus* (results not shown). The two species *Ammodytes tobianus* and *Hyperoplus lanceolatus* did not show any diagnostic characters on species level. Consequently, only two of the four investigated species can be identified using diagnostic characters on the basis of COI barcode sequences.

### Nc DNA analysis

The nc Rhodopsin sequence alignment showed a length of 464 bp after primer trimming. The complete fragment could be amplified and sequenced for all 70 specimens used for the mt DNA barcode analysis. The number of variable sites was very low and the alignment could be easily evaluated by eye. One diagnostic character was found for each of the species *Ammodytes marinus* (Table 5; site 460: C instead of A), *Hyperoplus lanceolatus* (site 82: T instead of C), and *Hyperoplus immaculatus* (site 433: A instead of G). *Ammodytes tobianus* showed no species specific mutation but could be characterised by a combination of all tree variable sites (Table 5, underlined bases). The Rhodopsin sequences from GenBank were compared with our sequences; the two *Hyperoplus lanceolatus* sequences (GenBank accessions: EU492010, EU492011) showed concordant results. In the case of the *Ammodytes tobianus* sequence (GenBank accession: AY141306) no data was available for site 460 but the two other sites were in agreement with our results.

**Table 5.** Variable sites identified for the nc Rhodopsin gene fragment sequence alignment. Bases in bold indicate species specific diagnostic characters. The three underlined bases are distinctive for *Ammodytes tobianus*.

		Nucleotide position		
Species	Specimens	82	433	460
*Ammodytes marinus*	27	C	G	**C**
*Ammodytes tobianus*	7*	C	G	A
*Hyperoplus lanceolatus*	30**	**T**	G	A
*Hyperoplus immaculatus*	8	C	**A**	A

*one /**two sequences downloaded from GenBank.

## Discussion

### Identification of genera and species using morphological characters

The primary objective of this study was to contribute to robust genera- and species-level identifications, combining morphological and molecular methods, of four closely related species of sand lances of the genera *Ammodytes* and *Hyperoplus* occurring in the northeast Atlantic Ocean and adjacent waters.

The detailed morphological analyses confirmed findings described by other authors (e.g. Duncker and Mohr 1939, Reay 1986): the genus *Ammodytes* can be distinguished by two morphological characters from the genus *Hyperoplus*. *Ammodytes* has clear protrusible premaxillae and no vomerine teeth. In contrast, *Hyperoplus* has no clear protrusible premaxillae and a pair of vomerine teeth. It should be noted here that Kayser (1961) found out that *Hyperoplus* has no real vomerine teeth, but anterior hooked ends of the prevomer instead.

Subsequently, Ida et al. (1994) pointed out that the tip of the prevomer in *Ammodytes* is straight, not protruded from the roof of the mouth, whereas in *Hyperoplus* the tip of the prevomer curved downwards, protruding from the roof of the mouth. According to Wiecaszek et al. (2007), the genus *Ammodytes* also has a longer lower jaw when compared to the length of pectoral-fin, while this relationship is reversed in *Hyperoplus*.

This study adds three more characters helpful in distinguishing between both genera of sand lances based on the four species considered. Firstly, the number of dermal plicae is significantly higher in *Hyperoplus* compared to *Ammodytes*. Secondly, *Hyperoplus* has a lower pectoral-fin length in relation to standard length (SL) than *Ammodytes*. Goltberg (1910) also reported a lower value of pectoral-fin length expressed as a proportion of head length for *Hyperoplus lanceolatus* than for *Ammodytes tobianus*. Thirdly, *Hyperoplus* has a larger mean snout length in % SL than *Ammodytes*. However, the last mentioned character is less recommended for practical taxonomical assignments, since its ranges overlap between the genera to a relatively large extent. Therefore, a combination of the following four characters remains, which seems to be useful to distinguish between the genera *Hyperoplus* and *Ammodytes*: protrusibility of premaxillae, presence of the hooked ends of prevomer, number of dermal plicae, and pectoral-fin length in % SL.

As indicated by the results of discriminant function analysis, morphometric measurements seem not to be characters of the first choice to distinguish the two species of each of the two genera, since they could not be discriminated by the first discriminant function.

According to the results presented here, six meristic characters (the number of lower arch gill rakers, the total number of gill rakers, the number of caudal vertebrae, the number of total vertebrae, and the number of dorsal-fin and anal-fin rays) are more useful than morphometric measurements to distinguish between *Hyperoplus immaculatus* and *Hyperoplus lanceolatus*. The use of these additional characters would support and refine the current methods to separate *Hyperoplus lanceolatus* from *Hyperoplus immaculatus*. Searching only for the occurrence of a conspicuous dark spot on either side of snout below anterior nostril could be unsuccessful in the case of preserved specimens.

In the case of *Ammodytes tobianus* and *Ammodytes marinus*, these results support the information on useful distinguishing characters between both species reported for instance by Reay (1986): *Ammodytes tobianus* differs from *Ammodytes marinus* by its belly scales that are organised in tight chevrons, scales which are present over musculature at base of caudal fin, as well as by lower numbers of dermal plicae, dorsal-fin rays and vertebrae. It should be mentioned that our analyses included also *Ammodytes tobianus* from the Baltic Sea, for which no meristic or morphometric data had been published, except for the number of vertebrae and pectoral-fin length (Wiecaszek et al. 2007).

### Discrimination of genera and species based on molecular data

The successful discrimination of the two sand lance species *Ammodytes marinus* and *Hyperoplus immaculatus* by DNA barcoding was already demonstrated by Knebelsberger et al. (2014) and could be confirmed by the present study. An additional three specimens of *Ammodytes marinus* were added to the dataset and the NJ analysis revealed well-supported monophyletic species clusters for *Ammodytes marinus* and *Hyperoplus immaculatus*, indicating an unambiguous separation of these two species. Successful species discrimination can also be demonstrated by the presence of gaps between intra- and interspecific genetic distances (Meyer and Paulay 2005), which were in case of *Ammodytes marinus* and *Hyperoplus immaculatus* 2.73 and 3.34 respectively. The BIN analysis performed on BOLD revealed two separate species BINs: one concordant BIN exclusively contained sequences which were taxonomically annotated as *Ammodytes marinus*, and a second discordant BIN contained all specimens of *Hyperoplus lanceolatus* and two further entries referring to as *Ammodytes marinus*. These two individuals were provided by other sources and may represent cases of misidentification, as all other *Ammodytes marinus* entries appeared in the concordant BIN.

Surprisingly, the two species *Ammodytes tobianus* and *Hyperoplus lanceolatus* belonging to different genera cannot be clearly separated on the basis of genetic distances, as the lowest distance (K2P) between these two species was only 0.15% and within species variation was found to be 0.15 and 0.62% respectively. In the NJ dendrogram both species appeared together in a well supported clade and were also found within the same BIN cluster when analysed together with data on BOLD. However, *Ammodytes tobianus* and *Hyperoplus lanceolatus* do not show haplotype sharing, as *Ammodytes tobianus* sequences appeared together in a separate cluster. The two species may therefore be separated by applying tree-based approaches like GMYC or model-based ones like ABGD.

In contrast to the barcoding results, both genera of *Ammodytes* and *Hyperoplus* can undoubtedly be separated by morphological character traits as discussed above. DNA barcoding failure between closely related congeneric species is usually more common than between species belonging to different genera (e.g. McCusker et al. 2013, Knebelsberger et al. 2015). For congeneric species of the genus *Ammodytes* inconsistencies between morphological data and DNA barcodes have already been demonstrated. For instance *Ammodytes americanus* DeKay, 1842 and *Ammodytes dubius* Reinhardt, 1837 from the northwest Atlantic Ocean could not be separated by DNA barcoding, possibly caused by inadequate taxonomy (McCusker et al. 2013), which may also concern the two species *Ammodytes personatus* Girard, 1856 and *Ammodytes hexapterus* Pallas, 1814 from the north Pacific (Turanov and Kartavtsev 2014).

In the present work, inadequate taxonomy, erroneous species designation or identification error can be excluded as possible explanation for DNA barcoding failure in unambiguously separating *Ammodytes tobianus* from *Hyperoplus lanceolatus*. In addition, true biological phenomena such as the occurrence of hybridisation or incomplete lineage sorting seem to be unlikely, as no interspeciﬁc haplotype sharing was found. In cases where mitochondrial COI sequences fail to distinguish between species, the application of nuclear DNA markers may be tested alternatively. In fish, the nuclear Rhodopsin gene has already been proposed as supplementary marker in order to identify species (Sevilla et al. 2007). However, most studies demonstrated reduced species discrimination success using nuclear Rhodopsin sequences compared to *COI* barcodes (Hanner et al. 2011, Collins et al. 2012, Behrens-Chapuis et al. 2015). In our study, the analysis of a short nuclear Rhodpsin gene fragment revealed diagnostic nucleotides for the species *Ammodytes marinus*, *Hyperoplus lanceolatus* and *Hyperoplus immaculatus*. The species *Ammodytes tobianus* can be characterised by the lack of species specific mutations compared to the other three species. Consequently, all four species of sand lances can be identified using the diagnostic character approach in combination with nuclear Rhodopsin sequences. In contrast to that, *COI* provided diagnostic characters only for the two species *Ammodytes marinus* and *Hyperoplus immaculatus*. *Ammodytes tobianus* and *Hyperoplus lanceolatus* cannot be characterised by this approach.

Our study clearly demonstrated that nuclear Rhodopsin constitutes a preferable alternative marker to discriminate successfully between the four investigated species of sand lances.

Finally, it should be pointed out that the present results are not meant to provide a phylogenetic reconstruction with regard to the genera *Ammodytes* and *Hyperoplus*, since the latter requires a more detailed study of more species of both genera, as well as other members of the group. However, accurate identification of these sand lance species is the basis to assess the status of their stocks and to implement appropriate measures of fisheries management or conservation, and as such, the aim of successfully identifying the NE Atlantic species has been accomplished.

## Conclusion

With this study a robust genus- and species-level discrimination of the four most abundant and closely related species of sand lances of the genera *Ammodytes* and *Hyperoplus* in the NE Atlantic Ocean and adjacent waters has been provided. It is expected that these results will facilitate the accurate identification of *Ammodytes marinus*, *Ammodytes tobianus*, *Hyperoplus immaculatus*, and *Hyperoplus lanceolatus* combining morphological and molecular methods.

## Supplementary materials

Supplementary material 1 Table S1 Data type: specimen data Explanation note: Supplementary metadata for specimens used for both morphological and genetic analyses; Museum and Sample IDs are specimen identifiers, BOLD Process IDs are unique codes automatically generated for each record on BOLD, GenBank Accession NOs represent sequence identifiers. This dataset is made available under the Open Database License (http://opendatacommons.org/licenses/odbl/1.0/). The Open Database License (ODbL) is a license agreement intended to allow users to freely share, modify, and use this Dataset while maintaining this same freedom for others, provided that the original source and author(s) are credited.Ralf Thiel, Thomas Knebelsberger

Supplementary material 2 Table S2 Data type: specimen data Explanation note: Museum IDs and collection data for specimens of *Ammodytes tobianus* used for morphological analyses only. This dataset is made available under the Open Database License (http://opendatacommons.org/licenses/odbl/1.0/). The Open Database License (ODbL) is a license agreement intended to allow users to freely share, modify, and use this Dataset while maintaining this same freedom for others, provided that the original source and author(s) are credited.Ralf Thiel, Thomas Knebelsberger
