# Supplementary material for: How reliably can northeast Atlantic sand lances of the genera Ammodytes and Hyperoplus be distinguished? A comparative application of morphological and molecular methods
Source: Zookeys. 2016 Sep 15;(617):139–64. doi: 10.3897/zookeys.617.8866 (PMC5027774; doi:10.3897/zookeys.617.8866)
Supplement: Supplementary material 1 — Table S1 [file zookeys-617-139-s001.docx]

**Supplementary material:**

**Table S1.** Supplementary Metadata for specimens used for both morphological and genetic analyses.

| **Identification** | **Museum ID** | **Sample ID** | **BOLD Process ID** | **GenBank Accession NO. COI** | **GenBank Accession NO. Rhodopsin** |
| --- | --- | --- | --- | --- | --- |
| *Ammodytes marinus* | ZMH 200091 | MT03009 | BNSF607-12 | ##### | ##### |
| *Ammodytes marinus* | ZMH 200092 | MT03010 | BNSF608-12 | ##### | ##### |
| *Ammodytes marinus* | ZMH 200093 | MT03011 | BNSF609-12 | ##### | ##### |
| *Ammodytes marinus* | ZMH 200103 | MT04209 | BNSFI097-12 | ##### | ##### |
| *Ammodytes marinus* | ZMH 200108 | MT04214 | BNSFI093-12 | ##### | ##### |
| *Ammodytes marinus* | ZMH 200109 | MT04215 | BNSFI094-12 | ##### | ##### |
| *Ammodytes marinus* | ZMH 200110 | MT04216 | BNSFI095-12 | ##### | ##### |
| *Ammodytes marinus* | ZMH 200111 | MT04217 | BNSFI096-12 | ##### | ##### |
| *Ammodytes marinus* | ZMH 200113 | MT04219 | BNSFI102-12 | ##### | ##### |
| *Ammodytes marinus* | ZMH 200114 | MT04220 | BNSFI103-12 | ##### | ##### |
| *Ammodytes marinus* | ZMH 200115 | MT04221 | BNSFI104-12 | ##### | ##### |
| *Ammodytes marinus* | ZMH 200117 | MT04223 | BNSFI106-12 | ##### | ##### |
| *Ammodytes marinus* | ZMH 200131 | MT07684 | BNSSE011-15 | ##### | ##### |
| *Ammodytes marinus* | ZMH 200132 | MT07685 | BNSSE012-15 | ##### | ##### |
| *Ammodytes marinus* | ZMH 200133 | MT07686 | BNSSE013-15 | ##### | ##### |
| *Ammodytes marinus* | ZMH 200170 | MT01849 | BNSF177-11 | ##### | ##### |
| *Ammodytes marinus* | ZMH 200171 | MT01850 | BNSF178-11 | ##### | ##### |
| *Ammodytes marinus* | ZMH 200172 | MT01851 | BNSF179-11 | ##### | ##### |
| *Ammodytes marinus* | ZMH 200173 | MT01852 | BNSF180-11 | ##### | ##### |
| *Ammodytes marinus* | ZMH 200174 | MT01853 | BNSSE001-15 | ##### | ##### |
| *Ammodytes marinus* | ZMH 200175 | MT01854 | BNSSE002-15 | ##### | ##### |
| *Ammodytes marinus* | ZMH 200178 | MT01910 | BNSF345-11 | ##### | ##### |
| *Ammodytes marinus* | ZMH 200185 | MT02952 | BNSF531-12 | ##### | ##### |
| *Ammodytes marinus* | ZMH 200187 | MT02954 | BNSF533-12 | ##### | ##### |
| *Ammodytes marinus* | ZMH 200188 | MT02998 | BNSF596-12 | ##### | ##### |
| *Ammodytes marinus* | ZMH 200189 | MT03007 | BNSF605-12 | ##### | ##### |
| *Ammodytes marinus* | ZMH 200190 | MT03008 | BNSF606-12 | ##### | ##### |
| *Ammodytes tobianus* | ZMH 200105 | MT04211 | BNSFI099-12 | ##### | ##### |
| *Ammodytes tobianus* | ZMH 200106 | MT04212 | BNSFI100-12 | ##### | ##### |
| *Ammodytes tobianus* | ZMH 200107 | MT04213 | BNSFI092-12 | ##### | ##### |
| *Ammodytes tobianus* | ZMH 200112 | MT04218 | BNSFI101-12 | ##### | ##### |
| *Ammodytes tobianus* | ZMH 200120 | MT05383 | BNSFI003-12 | ##### | ##### |
| *Ammodytes tobianus* | ZMH 200121 | MT05384 | BNSFI004-12 | ##### | ##### |
| *Hyperoplus immaculatus* | ZMH 200101 | MT04127 | BNSFI084-12 | ##### | ##### |
| *Hyperoplus immaculatus* | ZMH 200102 | MT04128 | BNSFI085-12 | ##### | ##### |
| *Hyperoplus immaculatus* | ZMH 200179 | MT02299 | BNSF255-11 | ##### | ##### |
| *Hyperoplus immaculatus* | ZMH 200180 | MT02887 | BNSF466-12 | ##### | ##### |
| *Hyperoplus immaculatus* | ZMH 200181 | MT02888 | BNSF467-12 | ##### | ##### |
| *Hyperoplus immaculatus* | ZMH 200182 | MT02889 | BNSF468-12 | ##### | ##### |
| *Hyperoplus immaculatus* | ZMH 200183 | MT02890 | BNSF469-12 | ##### | ##### |
| *Hyperoplus immaculatus* | ZMH 200184 | MT02891 | BNSF470-12 | ##### | ##### |
| *Hyperoplus lanceolatus* | ZMH 200094 | MT03041 | BNSF639-12 | ##### | ##### |
| *Hyperoplus lanceolatus* | ZMH 200095 | MT03042 | BNSF640-12 | ##### | ##### |
| *Hyperoplus lanceolatus* | ZMH 200096 | MT03043 | BNSF641-12 | ##### | ##### |
| *Hyperoplus lanceolatus* | ZMH 200097 | MT03044 | BNSF642-12 | ##### | ##### |
| *Hyperoplus lanceolatus* | ZMH 200098 | MT03045 | BNSF643-12 | ##### | ##### |
| *Hyperoplus lanceolatus* | ZMH 200099 | MT04125 | BNSFI079-12 | ##### | ##### |
| *Hyperoplus lanceolatus* | ZMH 200100 | MT04126 | BNSFI080-12 | ##### | ##### |
| *Hyperoplus lanceolatus* | ZMH 200118 | MT05381 | BNSFI001-12 | ##### | ##### |
| *Hyperoplus lanceolatus* | ZMH 200119 | MT05382 | BNSFI002-12 | ##### | ##### |
| *Hyperoplus lanceolatus* | ZMH 200122 | MT05385 | BNSFI005-12 | ##### | ##### |
| *Hyperoplus lanceolatus* | ZMH 200123 | MT07676 | BNSSE003-15 | ##### | ##### |
| *Hyperoplus lanceolatus* | ZMH 200124 | MT07677 | BNSSE004-15 | ##### | ##### |
| *Hyperoplus lanceolatus* | ZMH 200125 | MT07678 | BNSSE005-15 | ##### | ##### |
| *Hyperoplus lanceolatus* | ZMH 200126 | MT07679 | BNSSE006-15 | ##### | ##### |
| *Hyperoplus lanceolatus* | ZMH 200127 | MT07680 | BNSSE007-15 | ##### | ##### |
| *Hyperoplus lanceolatus* | ZMH 200128 | MT07681 | BNSSE008-15 | ##### | ##### |
| *Hyperoplus lanceolatus* | ZMH 200129 | MT07682 | BNSSE009-15 | ##### | ##### |
| *Hyperoplus lanceolatus* | ZMH 200130 | MT07683 | BNSSE010-15 | ##### | ##### |
| *Hyperoplus lanceolatus* | ZMH 200134 | MT07697 | BNSSE014-15 | ##### | ##### |
| *Hyperoplus lanceolatus* | ZMH 200135 | MT07698 | BNSSE015-15 | ##### | ##### |
| *Hyperoplus lanceolatus* | ZMH 200136 | MT07699 | BNSSE016-15 | ##### | ##### |
| *Hyperoplus lanceolatus* | ZMH 200137 | MT07700 | BNSSE017-15 | ##### | ##### |
| *Hyperoplus lanceolatus* | ZMH 200138 | MT08401 | BNSSE018-15 | ##### | ##### |
| *Hyperoplus lanceolatus* | ZMH 200139 | MT08402 | BNSSE019-15 | ##### | ##### |
| *Hyperoplus lanceolatus* | ZMH 200140 | MT08403 | BNSSE020-15 | ##### | ##### |
| *Hyperoplus lanceolatus* | ZMH 200141 | MT08404 | BNSSE021-15 | ##### | ##### |
| *Hyperoplus lanceolatus* | ZMH 200142 | MT08405 | BNSSE022-15 | ##### | ##### |
| *Hyperoplus lanceolatus* | ZMH 200176 | MT01855 | BNSF181-11 | ##### | ##### |
| *Hyperoplus lanceolatus* | ZMH 200177 | MT01856 | BNSF182-11 | ##### | ##### |
