# Supplementary material for: How reliably can northeast Atlantic sand lances of the genera Ammodytes and Hyperoplus be distinguished? A comparative application of morphological and molecular methods
Source: Zookeys. 2016 Sep 15;(617):139–64. doi: 10.3897/zookeys.617.8866 (PMC5027774; doi:10.3897/zookeys.617.8866)
Supplement: Supplementary material 2 — Table S2 [file zookeys-617-139-s002.docx]

**Table S2.** Supplementary Metadata for specimens of *Ammodytes tobianus* used for morphological analyses only.

| **Museum ID** | **Collection Date** | **Latitude** | **Longitude** | **Exact Side** | **Depth** | **Vessel** | **Trawl Type** | **Site Code** |
| --- | --- | --- | --- | --- | --- | --- | --- | --- |
| ZMH 26097-1 | 14.10.2003 | 54°27.945´N | 14° 31.272´E | Baltic Sea, Oderbank | 10,9 | Strelasund | Hopper Trawl | Station OB10, Haul 9 |
| ZMH 26097-2 | 14.10.2003 | 54°27.945´N | 14° 31.272´E | Baltic Sea, Oderbank | 10,9 | Strelasund | Hopper Trawl | Station OB10, Haul 9 |
| ZMH 26097-3 | 14.10.2003 | 54°27.945´N | 14° 31.272´E | Baltic Sea, Oderbank | 10,9 | Strelasund | Hopper Trawl | Station OB10, Haul 9 |
| ZMH 26097-4 | 14.10.2003 | 54°27.945´N | 14° 31.272´E | Baltic Sea, Oderbank | 10,9 | Strelasund | Hopper Trawl | Station OB10, Haul 9 |
| ZMH 26097-5 | 14.10.2003 | 54°27.945´N | 14° 31.272´E | Baltic Sea, Oderbank | 10,9 | Strelasund | Hopper Trawl | Station OB10, Haul 9 |
| ZMH 26098-1 | 08.07.2004 | 54°22.970´N | 14° 29.675´E | Baltic Sea, Oderbank | 9,3 | Strelasund | Hopper Trawl | Station OB07, Haul 49 |
| ZMH 26098-2 | 08.07.2004 | 54°22.970´N | 14° 29.675´E | Baltic Sea, Oderbank | 9,3 | Strelasund | Hopper Trawl | Station OB07, Haul 49 |
| ZMH 26098-3 | 08.07.2004 | 54°22.970´N | 14° 29.675´E | Baltic Sea, Oderbank | 9,3 | Strelasund | Hopper Trawl | Station OB07, Haul 49 |
| ZMH 26098-4 | 08.07.2004 | 54°22.970´N | 14° 29.675´E | Baltic Sea, Oderbank | 9,3 | Strelasund | Hopper Trawl | Station OB07, Haul 49 |
| ZMH 26098-5 | 08.07.2004 | 54°22.970´N | 14° 29.675´E | Baltic Sea, Oderbank | 9,3 | Strelasund | Hopper Trawl | Station OB07, Haul 49 |
| ZMH 26099-1 | 12.10.2004 | 54°03.451´N | 14° 06.516´E | Baltic Sea, Usedom coastal waters | 13,4 | Strelasund | Hopper Trawl | Station KG18, Haul 64 |
| ZMH 26099-2 | 12.10.2004 | 54°03.451´N | 14° 06.516´E | Baltic Sea, Usedom coastal waters | 13,4 | Strelasund | Hopper Trawl | Station KG18, Haul 64 |
| ZMH 26099-3 | 12.10.2004 | 54°03.451´N | 14° 06.516´E | Baltic Sea, Usedom coastal waters | 13,4 | Strelasund | Hopper Trawl | Station KG18, Haul 64 |
| ZMH 26099-4 | 12.10.2004 | 54°03.451´N | 14° 06.516´E | Baltic Sea, Usedom coastal waters | 13,4 | Strelasund | Hopper Trawl | Station KG18, Haul 64 |
| ZMH 26099-5 | 12.10.2004 | 54°03.451´N | 14° 06.516´E | Baltic Sea, Usedom coastal waters | 13,4 | Strelasund | Hopper Trawl | Station KG18, Haul 64 |
